# Supplementary material for: Polysome-CAGE of TCL1-driven chronic lymphocytic leukemia revealed multiple N-terminally altered epigenetic regulators and a translation stress signature
Source: eLife. 2022 Aug 8;11:e77714. doi: 10.7554/eLife.77714 (PMC9359700; doi:10.7554/eLife.77714)
Supplement: Figure 3—source data 1. [file elife-77714-fig3-data1.pptx]

## Slide 1
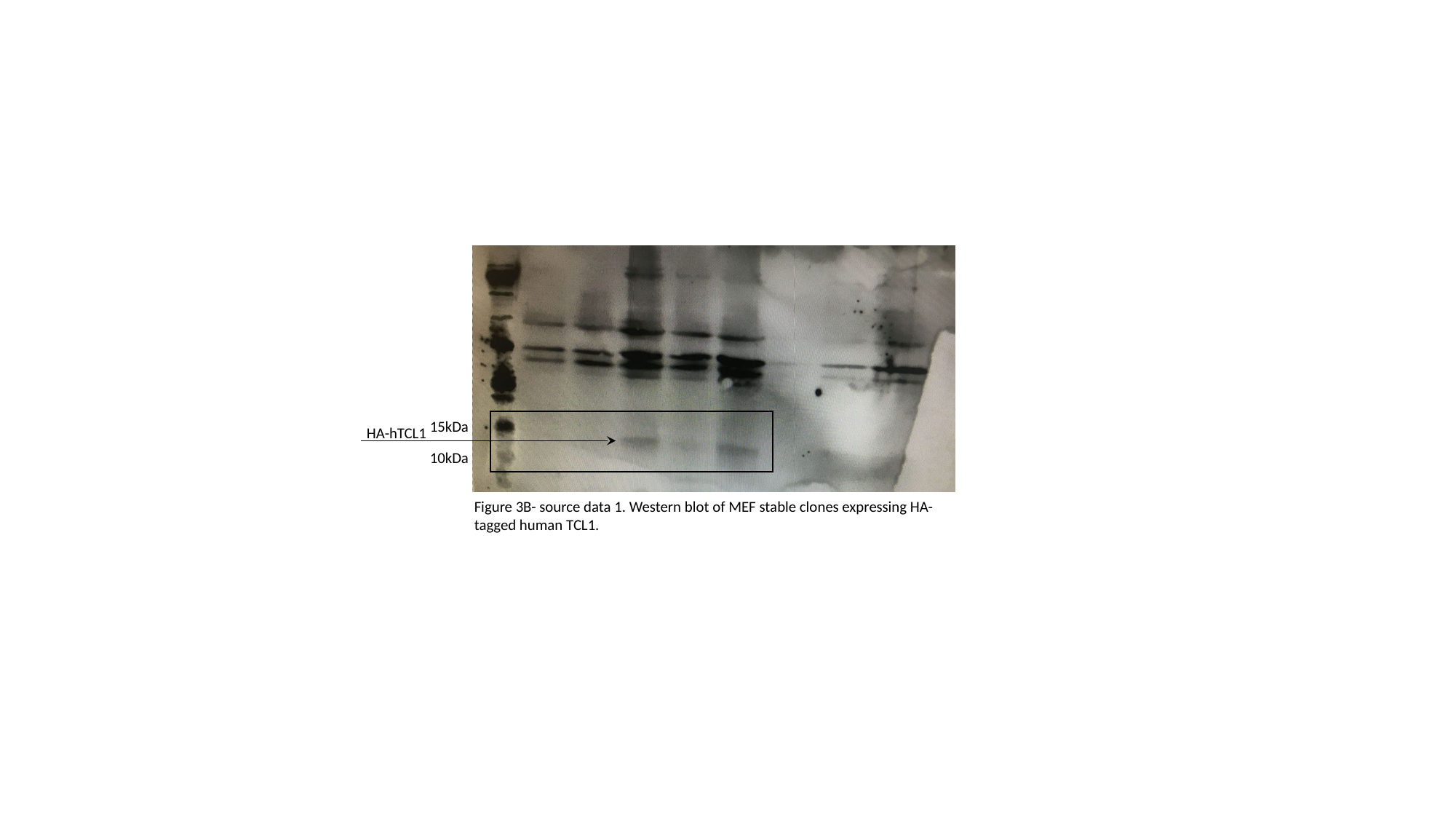

15kDa
HA-hTCL1
10kDa
Figure 3B- source data 1. Western blot of MEF stable clones expressing HA-tagged human TCL1.
