# Supplementary material for: Polysome-CAGE of TCL1-driven chronic lymphocytic leukemia revealed multiple N-terminally altered epigenetic regulators and a translation stress signature
Source: eLife. 2022 Aug 8;11:e77714. doi: 10.7554/eLife.77714 (PMC9359700; doi:10.7554/eLife.77714)
Supplement: Supplementary file 1. — GO enrichment analysis by the GeneAnalytics tool of GeneCardSuite, performed over a total of 923 gene set affected by differential TSS usage, generating alternative isoforms either up- or downregulated in Eu-Tcl1 mice. SuperPath matching score is based on the binomial distribution. [file elife-77714-supp1.docx]

**Figure 2 supplementary table 1.** GO pathways affected by differential TSS usage.

| Super-pathways | | | | |
| --- | --- | --- | --- | --- |
| Score | SuperPath Name | Total Genes | Matched Genes | Matched Genes (Symbols) |
| 20.46 | NF-kappaB Signaling | 327 | 30 | NLRX1, TNIP1, TEC, PTK2B, LITAF, AIM2, ZAP70, IRAK2, PTPN22, BACH2, PTPRC, RUNX1, TLR1, TLR6, IRF5, NFATC1, TNFAIP3, MYB, ZBTB7B, TRAF5, IKZF3, TCF12, CIITA, IKZF1, RELA, CEBPB, SIVA1, FYB1, PDCD1LG2, NFATC2 |
| 19.02 | MAPK Pathway | 88 | 14 | CASP8, RPS6KA3, PIK3R1, PIK3R2, PTK2, PIK3R4, TLR1, TLR6, PIK3CD, RPS6KA1, IL5RA, PIK3CG, RELA, MAPK11 |
| 18.59 | RET Signaling | 971 | 60 | ABHD6, IQGAP1, RPS6KA3, AKT1S1, TEC, TNRC6B, PTK2B, LPAR5, VCL, PIK3R1, RAPGEF1, PIK3R2, MDM2, SPTBN1, PPP5C, CUL1, AKAP13, AGO3, ARHGEF2, PTK2, PIK3R4, ARHGEF3, TRIO, EEF2K, DUSP10, ARHGEF10, UBE2V1, RGS3, RTN4, BCL2L11, EPS15L1, MAP3K11, DGKZ, PSMD14, CSK, NFATC1, ITSN1, FGFR2, UBA52, CACNB1, RGS19, DGKE, PIK3CD, RPS6KA1, IL5RA, RASGRP1, CDKN1A, GNG10, PXN, ELMO1, NF1, RASAL1, SH2D2A, LPAR6, RASGRP2, RELA, RHEB, MAPK11, DAB2IP, NFATC2 |
| 18.47 | DNA Damage | 296 | 27 | RCC2, MDM2, PPP5C, POLG, ANP32A, MGMT, WWOX, MASTL, MCPH1, TACC3, AURKB, BRD2, S100A6, WEE1, CDC45, RBBP8, SMARCA5, RECQL5, CDKN1A, CDKN2C, XRCC1, NUDT1, FOXM1, RPA1, MCM7, NEK7, NBN |
| 15.44 | Cellular Senescence (REACTOME) | 452 | 33 | RPS6KA3, AKT1S1, TNRC6B, TXNRD1, MDM2, P4HB, DNAJC7, E2F3, E2F2, AGO3, EGLN2, PIK3R4, ARNT, MTMR14, PSMD14, H3C3, UBA52, EHMT2, RPS6KA1, H2BU1, PHC2, CDKN1A, CDKN2C, RELA, CEBPB, ERO1A, RHEB, RPA1, MAPK11, CUL2, NBN, NDC1, H2BC9 |
| 15.20 | Regulation of RhoA Activity | 46 | 9 | AKAP13, ARHGAP35, ARHGEF2, ARHGEF3, TRIO, ARAP1, ARHGAP4, ARHGEF10, ARHGAP9 |
| 14.26 | AMP-activated Protein Kinase (AMPK) Signaling | 167 | 17 | AKT1S1, PIK3R1, PIK3R2, HDAC5, PTK2, SCD, EEF2K, PFKFB4, PIK3CD, RPS6KA1, TBC1D1, CDKN1A, HDAC4, CAMKK1, SREBF1, PIK3CG, RHEB |
| 14.21 | Cell Cycle, Mitotic | 622 | 40 | AHCTF1, RCC2, DSN1, MDM2, E2F3, CUL1, E2F2, KIF20A, SET, PSMD14, MASTL, MCPH1, H3C3, AURKB, UBA52, PMF1, MDC1, LIN52, WEE1, FBXO5, H2BU1, CDC45, RBBP8, CDK5RAP2, SYNE2, SMARCA5, LIN54, CDKN1A, GINS1, CDKN2C, RUVBL2, MSH5, FOXM1, LPIN2, RPA1, MCM7, NEK7, NBN, NDC1, H2BC9 |
| 13.21 | Oncogene Induced Senescence | 32 | 7 | TNRC6B, MDM2, E2F3, E2F2, AGO3, UBA52, CDKN2C |
| 12.35 | Apoptosis Pathway | 152 | 15 | CASP8, RPS6KA3, PIK3R1, PIK3R2, MDM2, PTK2, PIK3R4, FGFR2, CAPN1, PIK3CD, RPS6KA1, IL5RA, PIK3CG, RELA, MAPK11 |
| 12.33 | Class I MHC Mediated Antigen Processing and Presentation | 823 | 47 | KIF3C, AKT1S1, TNRC6B, PIK3R1, PIK3R2, MDM2, ZAP70, TRIM11, KLRB1, CUL1, AGO3, MAP3K8, PTPN22, FBXL19, FBXO7, UBE2V1, KIF20A, RNF123, PTPRC, TLR1, TLR6, PSMD14, KLC3, CSK, FGFR2, SIAH2, CUL7, UBA52, RAP1GAP2, UBA1, RAPGEF3, PIK3CD, RASGRP1, UBE2M, CDKN1A, DZIP3, KEAP1, RAPGEF4, HLA-DOB, RASGRP2, RELA, BTLA, STIM1, FBXL3, FYB1, PDCD1LG2, CUL2 |
| 12.25 | B Cell Receptor Signaling Pathway (KEGG) | 153 | 15 | TEC, PIK3R1, RAPGEF1, PIK3R2, E2F3, SH3BP2, PTPRC, CSK, NFATC1, PIK3CD, RPS6KA1, PIK3CG, RELA, MEF2D, NFATC2 |
| 10.35 | Retinoblastoma (RB) in Cancer | 88 | 10 | MDM2, E2F3, E2F2, WEE1, CDC45, CDKN1A, HMGB1, RPA1, SUV39H1, MCM7 |
| GO | | | | |
| 22.96 | Guanyl-nucleotide Exchange Factor Activity | 219 | 25 | RCC2, SPATA13, DENND6B, RAPGEF1, ALS2, DOCK10, AKAP13, ARHGEF2, ARHGEF3, DOCK8, TRIO, PSD4, ARHGEF10, DENND2D, CYTH1, ITSN1, RGL2, RAPGEF3, RASGRP1, ELMO1, RAPGEF4, RASGRP2, EPS8, RIN3, MADD |
| 21.36 | Positive Regulation of Transcription By RNA Polymerase II | 1157 | 71 | IL18, RPS6KA3, TNIP1, SSBP4, ZBTB38, TCF20, NCOA7, ZNF76, LITAF, ZNF219, ZMIZ2, PIK3R1, PIK3R2, MDM2, E2F3, HDAC5, E2F2, MAFG, ARHGEF2, DLL1, RXRB, ARNT, SSBP3, ZMIZ1, ZNF296, WWOX, MAML2, PBX3, UBP1, RUNX1, ATF5, ZNF91, IRF5, COQ7, NFATC1, FGFR2, SIRT2, UBA52, LDB1, MYB, ZBTB7B, DBP, TCF4, RPS6KA1, IKZF3, TFE3, RREB1, TCF12, SMARCA5, ARNTL, CBFB, POU2F1, TET3, CCPG1, SETX, CHD7, HMGB1, HDAC4, NFIC, RUVBL2, GTF2F1, PPRC1, SREBF1, CIITA, RELA, CEBPB, FOXM1, LPIN2, MEF2D, DAB2IP, NFATC2 |
| 14.99 | B Cell Activation | 36 | 8 | CASP8, ZAP70, HDAC5, HSPD1, PIK3CD, IKZF3, RASGRP1, HDAC4 |
| 14.23 | Chromatin Remodeling | 104 | 13 | INO80E, HDAC5, PADI4, BAZ2B, ARID1B, MYB, BAZ2A, SMARCA5, RERE, CHD7, HDAC4, RUVBL2, KDM4A, CHD1, CHD9 |
| 14.14 | Cellular Response to DNA Damage Stimulus | 516 | 35 | ACTR5, INO80E, WDR76, ZBTB38, SIRT4, CYREN, PPP5C, APLF, PSMD14, MASTL, RAD51B, UBA1, SAMHD1, MDC1, GTF2H1, FOXP1, RBBP8, RECQL5, CDKN1A, GEN1, SETX, ZMPSTE24, XRCC1, HMGB1, RUVBL2, MSH5, FOXM1, RPA1, SUV39H1, MCM7, GNL1, NBN, NFATC2, FANCF |
| 13.84 | Phosphatidylinositol Biosynthetic Process | 78 | 11 | INPP5K, PIK3R1, PIK3R2, PIK3R4, MTMR14, MTMR4, INPP4B, DGKE, PIK3CD, PITPNM2, PIK3CG |
| 13.56 | Phosphorylation | 702 | 43 | SGMS1, RPS6KA3, TEC, PTK2B, ITPK1, ZAP70, HK1, PKN3, TNK2, SPHK2, MAP3K8, PTK2, PIK3R4, TRIO, EEF2K, SEPHS1, MAP3K11, SRPK2, DGKZ, CIT, MASTL, PHKG2, PKN1, CSK, AURKB, FGFR2, N4BP2, PFKFB4, DGKE, PIK3CD, WEE1, RPS6KA1, SGK3, CDKN1A, CDKN2C, RPS6KC1, CAMKK1, CDC42BPG, PIK3CG, CIITA, MAPK11, MARK4, NEK7 |
| 12.03 | Chromatin Organization | 345 | 25 | HDAC5, PADI4, ATXN7L3, ING1, BANP, H3C3, ARID1B, PKN1, BRD2, EHMT2, NSD1, BAZ2A, SMARCA5, WIZ, TET3, CHD7, ZMPSTE24, DNMT3A, CHD9, HDAC4, RUVBL2, CHD1, IKZF1, RELA, KDM4A, SUV39H1, ZCWPW1 |
| 12.01 | Chromatin Silencing Complex | 9 | 4 | SIRT2, BAZ2A, SMARCA5, SMYD4, SUV39H1 |
| 11.70 | DNA Methylation | 27 | 6 | MGMT, ATF7IP, EHMT2, BAZ2A, DNMT3A, GATAD2A |
