# Supplementary material for: Polysome-CAGE of TCL1-driven chronic lymphocytic leukemia revealed multiple N-terminally altered epigenetic regulators and a translation stress signature
Source: eLife. 2022 Aug 8;11:e77714. doi: 10.7554/eLife.77714 (PMC9359700; doi:10.7554/eLife.77714)
Supplement: Supplementary file 2. — Enrichment analysis of MGI phenotypes performed by the GeneAnalytics tool of GeneCardSuite over a total of 923 gene set affected by differential transcription start site (TSS) usage, generating alternative isoforms, either up- or downregulated in Eu-Tcl1 mice. MGI phenotypes matching scores are based on the binomial distribution. [file elife-77714-supp2.docx]

**Figure 2 supplementary table 2.** MGI phenotypes affected by differential TSS usage.

| Score | Name | Total Genes | Matched Genes | Symbols |
| --- | --- | --- | --- | --- |
| 31.00 | Increased B Cell Number | 92 | 19 | TNIP1, APOE, ZAP70, SPHK2, PTPN22, DLL1, RGS3, BCL2L11, PTPRC, DGKZ, MCPH1, TNFAIP3, TNFRSF13B, RASGRP1, CDKN1A, CDKN2C, IKZF1, SEMA4D, NFATC2 |
| 20.68 | Thymus Hypoplasia | 118 | 17 | WIPF1, AFF1, ZFP36, MDM2, ZAP70, NDST1, PTPRC, RUNX1, TCF12, CBFB, CHD7, PNP, ZMPSTE24, PIK3CG, IKZF1, NBN, GFI1 |
| 19.13 | Enlarged Spleen | 254 | 25 | TNIP1, ZFP36, MDM2, PPP5C, E2F2, PTPN22, ING1, WWOX, BCL2L11, PTPRC, RUNX1, PSTPIP2, UNC13D, TNFAIP3, MYB, TNFRSF13B, IKZF3, CBFA2T3, RASGRP1, CDKN1A, CDKN2C, IKZF1, CEBPB, STIM1, NFATC2 |
| 17.97 | "decreased CD4-positive, Alpha Beta T Cell Number" | 136 | 17 | ZAP70, DLL1, DOCK8, FBXO7, PTPRC, RUNX1, DGKZ, SIRT2, ZBTB7B, IL5RA, RASGRP1, PNP, RUVBL2, PIK3CG, CIITA, SEMA4D, NBN |
| 16.05 | Abnormal Cytokine Secretion | 151 | 17 | LITAF, AIM2, MAP3K8, SEMA4A, DUSP10, PTPRC, TLR1, TLR6, PSTPIP2, UNC13D, TNFAIP3, ZBTB32, CIITA, NLRC4, STIM1, PDCD1LG2, GFI1 |
