# Supplementary material for: Polysome-CAGE of TCL1-driven chronic lymphocytic leukemia revealed multiple N-terminally altered epigenetic regulators and a translation stress signature
Source: eLife. 2022 Aug 8;11:e77714. doi: 10.7554/eLife.77714 (PMC9359700; doi:10.7554/eLife.77714)
Supplement: Supplementary file 3. [file elife-77714-supp3.docx]

**Figure 4 supplementary table 1.** Transcription factors upregulated in Eu-Tcl1

| **symbol** | **logFC** | **adj. P.Val** |
| --- | --- | --- |
| Ebf3 | 5.80 | 0.01 |
| Zbtb7c | 4.80 | 0.02 |
| Plagl1 | 4.51 | 0.04 |
| Klf5 | 3.65 | 0.06 |
| Zbtb32 | 3.44 | 0.01 |
| Myb | 3.37 | 0.01 |
| E2f8 | 3.32 | 0.02 |
| Batf3 | 3.12 | 0.04 |
| Klf1 | 3.09 | 0.03 |
| Nr1d1 | 2.70 | 0.04 |
| E2f7 | 2.65 | 0.04 |
| Mybl2 | 2.64 | 0.03 |
| Mafb | 2.62 | 0.06 |
| Nfil3 | 2.59 | 0.01 |
| Bhlhe41 | 2.54 | 0.01 |
| Ets2 | 2.45 | 0.01 |
| Atf3 | 2.44 | 0.01 |
| Arid3a | 2.35 | 0.01 |
| Tfdp1 | 2.33 | 0.02 |
| Rxra | 2.11 | 0.05 |
| Mef2b | 2.03 | 0.01 |
| E2f1 | 1.87 | 0.02 |
| E2f3 | 1.79 | 0.04 |
| E2f6 | 1.59 | 0.04 |
| Creb3l2 | 1.54 | 0.03 |
| Myc | 1.49 | 0.01 |
| Pbx3 | 1.35 | 0.03 |
| Maff | 1.30 | 0.05 |
| Maz | 1.23 | 0.02 |
