# Supplementary material for: Polysome-CAGE of TCL1-driven chronic lymphocytic leukemia revealed multiple N-terminally altered epigenetic regulators and a translation stress signature
Source: eLife. 2022 Aug 8;11:e77714. doi: 10.7554/eLife.77714 (PMC9359700; doi:10.7554/eLife.77714)
Supplement: Supplementary file 4. [file elife-77714-supp4.docx]

Supplementary table 4

| Oligo/Primer | Assay | Sequence |
| --- | --- | --- |
| 5’ RACE TSO | 5’ RACE-qPCR | GCTAATCATTGCAAGCAGTGGTATCAACGCAGAGTACATrGrGrG |
| 5’ RACE RT Dnmt3a | 5’ RACE-qPCR | CCCGTTTCCGTTTGCTGATGTA |
| 5’ RACE RT Chd1 | 5’ RACE-qPCR | TGTCAGCAGAGGCTAGGTTAATGC |
| 5’ RACE RT Kdm4a | 5’ RACE-qPCR | GGCGACCCTTGGTCTTCTTATTC |
| 5’ RACE RT Sirt2 | 5’ RACE-qPCR | TCCCATCCTCCTCAACCTACTT |
| qPCR F | 5’ RACE-qPCR | CATTGCAAGCAGTGGTATCAAC |
| qPCR ATSS Dnmt3a R | 5’ RACE-qPCR | CTGAGGAGGGATTAGGAGTT |
| qPCR ATSS Chd1 R | 5’ RACE-qPCR | CGAGTGCTCAGGTTGGAAAT |
| qPCR ATSS Kdm4a R | 5’ RACE-qPCR | ATGGATGGCTCAGTGGTTAAG |
| qPCR ATSS Sirt2 R | 5’ RACE-qPCR | CAGGACTCACCAGGCTTTAC |
| Canonical Hdac5 F | qPCR MEFs | CCCGTCCGTCTGTCTGTTAT |
| Canonical Hdac5 R | qPCR MEFs | CTGCAGGAAGCTGACGTTG |
| Intragenic Hdac5 F | qPCR MEFs | CTGGGAGGGAACACAGAGAG |
| Intragenic Hdac5 R | qPCR MEFs | TCCAAAGGTTCCAGGAGTTG |
| Canonical Dnmt3a F | qPCR MEFs | gggcttgacatcagggtcta |
| Canonical Dnmt3a R | qPCR MEFs | ACGGTTCTCCTCCTGTTCCT |
| Intragenic Dnmt3a F | qPCR MEFs | GCCAACTCCTAATCCCTCCT |
| Intragenic Dnmt3a R | qPCR MEFs | GGACAGAGAGACAGGCAGGT |
| Canonical Kdm4a F | qPCR MEFs | tcgccctcaagaaagacagt |
| Canonical Kdm4a R | qPCR MEFs | AAAGGTCATGATCCGAGCAC |
| Intragenic Kdm4a F | qPCR MEFs | cttctcccctcagctctcct |
| Intragenic Kdm4a R | qPCR MEFs | CTCATCTGCAACctgagcaa |
| Canonical Zswim F | qPCR MEFs | GAGCCGTTGTTGGACCTTAG |
| Canonical Zswim R | qPCR MEFs | AACACAATGCGCTTCTGAAC |
| Intragenic Zswim F | qPCR MEFs | gcgcctccacttgcattt |
| Intragenic Zswim R | qPCR MEFs | GCTCTCCCGGAAAATCACTT |
